# Schmallenberg Virus Infection in Dogs, France, 2012

**DOI:** 10.3201/eid1911.130464

**Published:** 2013-11

**Authors:** Corinne Sailleau, Cassandre Boogaerts, Anne Meyrueix, Eve Laloy, Emmanuel Bréard, Cyril Viarouge, Alexandra Desprat, Damien Vitour, Virginie Doceul, Catherine Boucher, Stéphan Zientara, Alexandra Nicolier, Dominique Grandjean

**Affiliations:** ANSES, Maisons-Alfort, France (C. Sailleau, E. Bréard, C. Viarouge, A. Desprat, D. Vitour, V. Doceul, S. Zientara);; Ecole Nationale Vétérinaire d’Alfort, Maisons-Alfort (C. Boogaerts, A. Meyrueix, E. Laloy, D. Grandjean);; Royal Canin, Aimargues, France (C. Boucher);; Vet Diagnostics, Lyon, France (A. Nicolier)

**Keywords:** Schmallenberg virus, viruses, dogs, France, congenital

**To the Editor:** In 2011, Schmallenberg virus (SBV) emerged in Europe (*1*); the virus spread into France in January 2012 (*2*). During January–March 2012, a total of >1,000 cases were reported in France, mainly in stillborn and newborn lambs with congenital malformations. 

In March 2012, neurologic disorders were detected in five 15-day-old puppies (Belgian shepherd) from a dog breeding kennel in northwestern France (Orne). We report data suggesting that these puppies were infected with SBV.

In June 2012, the kennel veterinarian contacted a veterinary school (Unité de Médecine de l’Elevage et du Sport Breeding and Sport Medicine Unit, Maisons-Alfort, France) after neurologic signs of ataxia, exotropia, a head tilt, and stunted growth were observed in a litter of 5 puppies. Four of the puppies had died at 5–6 weeks of age. The veterinarian collected blood samples from the surviving puppy at 3 months of age, and the puppy was euthanized for necropsy. Severe torticollis was observed during the necropsy, but no other macroscopic signs were detected. The brain, including the cerebellum; a part of the spine; and cerebrospinal fluid (CSF) were collected for further investigation. Specific PCR analyses for canine coronavirus, *Neospora caninum*, *Toxoplasma gondii*, and canine minute virus were performed on CSF; all test results were negative. The brain tissue was fixed in formalin and processed for histologic examination. Features of degenerative encephalopathy, including neuronal vacuolation, neuropil vacuolation, and minimal gliosis, were observed. 

Because some clinical signs were evocative of SBV infection and the puppy was born in an area where the virus was circulating actively in cattle and sheep, veterinarians decided to investigate SBV as a possible etiology. Serum samples from the 3-month-old puppy and the dam were tested by virus neutralization test (VNT), according to the protocol used for ruminant serum. The results were negative for the puppy but positive (titer 128) for the mother. Specific competitive SBV ELISA (IDVet, Montpellier, France) against the SBV N protein showed similar results.

Real-time reverse transcription PCR (RT-PCR) was performed (*3*) to detect the presence of the SBV genome in the cerebellum. Because the sample was paraffin-embedded, RNA was extracted from 5-µm sections, as described (*4*). All of the extracted cerebellum sections had positive test results (cycle threshold range 33−36); the extraction and PCR controls all showed negative results. To confirm these positive results, conventional RT-PCR was used to amplify a 573-nt sequence of the SBV S segment. The amplification product was sequenced, and a BLAST analysis was performed (www.ncbi.nlm.nih.gov/BLAST). An identity of 100% was obtained with the SBV small gene segment from a ruminant (GenBank accession no. KC108860). An immunohistochemical assay was also performed; the result was negative. 

The remaining 7 female dogs in the breeding kennel were tested for SBV in October 2012; 1 showed positive test results by VNT (titer 256), which confirmed that SBV was circulating in the kennel. This positive dam had a litter of puppies in December 2012, but no signs developed, and the puppies were not tested. In March 2013, repeat testing was done on serum samples from the 2 dogs that had shown positive results. Results for both animals were positive by VNT (titers 32 for the dam and 128 for the other dog) and ELISA.

Taken together, specific SBV antibodies in the mother and the SBV genome in her puppy suggest that these dogs experienced SBV infection. The absence of detectable SBV antibodies in the puppy in this investigation suggests that transplacental infection occurred before the onset of fetal immune competence. Maternal infection probably occurred in January or February 2012; entomologic monitoring conducted in France showed the presence of *Culicoides* spp. midges, a vector of SBV, during this period in northwestern France. In addition, because the puppies were born in March 2012 and SBV antibodies were still detectable in the mother in March 2013, the duration of SBV antibodies in dogs appears to be >1 year. In cattle and sheep, the SBV genome persists in an infected fetus and is detectable after birth by real-time RT-PCR, despite gestation length (*5*,*6*).

Few reports on orthobunyavirus infections in dogs are available. Two serologic studies from the United States (*7*) and Mexico (*8*) found antibodies against La Crosse virus, South River virus, and Jamestown Canyon virus in dogs. Two other reports described cases in which La Crosse virus was detected in canine littermates who had clinical encephalitis (*9*) or neurologic disorders (*10*).

It is unclear if the apparent SBV infection we detected in these dogs was an isolated event or if other cases occurred elsewhere but were not detected because they were not investigated. Further serologic and clinical surveys are needed to estimate SBV prevalence in dogs and the virus’ involvement in the occurrence of neurologic signs in puppies.
